# TCTP Is an Androgen-Regulated Gene Implicated in Prostate Cancer

**DOI:** 10.1371/journal.pone.0069398

**Published:** 2013-07-22

**Authors:** Mari Kaarbø, Margrethe L. Storm, Su Qu, Håkon Wæhre, Bjørn Risberg, Håvard E. Danielsen, Fahri Saatcioglu

**Affiliations:** 1 Department of Biosciences, University of Oslo, Oslo, Norway; 2 Institute for Medical Informatics, Oslo University Hospital, Oslo, Norway; 3 Division of Pathology, Oslo University Hospital, Oslo, Norway; 4 Center for Cancer Biomedicine, Oslo University Hospital, Oslo, Norway; 5 Department of Informatics, University of Oslo, Oslo, Norway; Queensland University of Technology, Australia

## Abstract

TCTP has been implicated in a plethora of important cellular processes related to cell growth, cell cycle progression, malignant transformation and inhibition of apoptosis. In addition to these intracellular functions, TCTP has extracellular functions and plays an important role in immune cells. TCTP expression was previously shown to be deregulated in prostate cancer, but its function in prostate cancer cells is largely unknown. Here we show that TCTP expression is regulated by androgens in LNCaP prostate cancer cells *in vitro* as well as human prostate cancer xenografts *in vivo*. Knockdown of *TCTP* reduced colony formation and increased apoptosis in LNCaP cells, implicating it as an important factor for prostate cancer cell growth. Global gene expression profiling in TCTP knockdown LNCaP cells showed that several interferon regulated genes are regulated by TCTP, suggesting that it may have a role in regulating immune function in prostate cancer. In addition, recombinant TCTP treatment increased colony formation in LNCaP cells suggesting that secreted TCTP may function as a proliferative factor in prostate cancer. These results suggest that TCTP may have a role in prostate cancer development.

## Introduction

Prostate cancer is the most frequently diagnosed non-cutaneous malignancy and the third leading cause of cancer-related deaths in men in western industrialized countries [1]. The importance of androgens for the development and progression of prostate cancer was shown early in the 20^th^ century. This resulted in significant focus on androgens and the receptor to which they bind, the androgen receptor (AR) [2], and androgen ablation therapy became the main line of therapy. Even though AR and androgen action are critically important aspects in prostate cancer, it has become evident that other signaling pathways, as well as non-genomic and genomic alterations, are involved in the development and progression of prostate cancer (reviewed in [3]).

Translationally controlled tumor protein (TCTP) is a multifaceted factor which is highly conserved in a number of species. It was originally discovered in a mouse sarcoma cell line as a protein regulated at the translational level [4]. TCTP has since been implicated in a number of important cellular processes, such as cell growth, malignant transformation and inhibition of apoptosis. TCTP is not found exclusively in tumor cells, but has a widespread expression profile that is not restricted to a specific tissue or cell type. However, TCTP expression is generally higher in tumors compared to corresponding normal tissue (reviewed in [5]).

TCTP has an anti-apoptotic role in a number of cell lines (reviewed in [6]). TCTP knockout mice are embryonically lethal with reduced number of cells and a higher incidence of apoptosis in the embryos, highlighting its importance in early development [7], [8]. In addition, TCTP has been shown to bind calcium [9]–[12]; this property may be linked to its anti-apoptotic activity as the concentration of free intracellular calcium is known to increase during apoptosis, triggering a sequence of events leading to cell death [13]. TCTP is engaged in a variety of protein-protein interactions and binds tubulin, Plk-1, p53 and the guanine nucleotide exchange factor Rheb, amongst others [14]. In addition, *TCTP* mRNA is highly structured and activates PKR, a protein kinase involved in the inflammatory response [15]. Although these studies offer plausible explanations for the many reported effects of TCTP, the exact mechanisms by which TCTP functions remain to be delineated.

TCTP is also a secreted protein with extracellular functions [16]. The secreted form of TCTP was originally identified by its ability to promote histamine release from basophils in a subset of allergic donors and thus named Histamine Releasing Factor (HRF) [17]. Additionally, TCTP stimulated B-cell proliferation, induced expression of IL-1, IL-6, and immunoglobulin production consistent with a role as a B-cell growth factor [16]. TCTP does not contain an N-terminal signal sequence typical for secreted proteins and is secreted through a non-classical pathway involving exosomes [18]. Interestingly, nanovesicles secreted from apoptotic endothelial cells that act in a paracrine fashion contain TCTP, further extending the modality of its extracellular action [19].

Recent studies have identified TCTP expression in the human prostate. TCTP was found to be expressed in prostatic tissues from men undergoing surgical adenomectomy for benign prostatic hyperplasia (BPH) and in cell lines derived from normal prostate, such as the cell line PWR-1E, and prostate cancer [12]. TCTP expression was also found to be higher than other calcium binding proteins (CBPs) in the human prostate. In addition, immunohistochemical analyses of normal prostate indicated that TCTP was mainly expressed by the secretory luminal cells and to a smaller degree by the basal cells. TCTP was also identified in prostatic fluids, which suggests that it may have an extracellular role in the prostate [12]. In addition, TCTP may affect proliferation and viability in prostate cancer cells [20]. LNCaP cells treated with siRNA targeting TCTP indicated that knockdown of TCTP increases apoptosis through caspase 3 and caspase 8 and reduces cell proliferation in LNCaP cells [20]. Furthermore, results from a recent study indicate that heat shock protein 27 (Hsp27) interacts with TCTP in prostate cancer cells and protects it from degradation [21]. In addition, TCTP expression levels correlated with disease progression, where it was upregulated in castration resistant prostate cancer (CRPC) [21]. These studies implicate TCTP in prostate cancer; however, there is limited information on its regulation and function in prostate cancer cells.

Here we show that TCTP is regulated by androgens, important survival factors for prostate cancer cells as well as the normal prostate. Ectopic expression of TCTP and its knockdown, as well as experiments with recombinant TCTP, show that it contributes to cell growth and proliferation, implicating it as an important factor in prostate cancer.

## Materials and Methods

### Ethics Statement

This study was approved by the Regional Ethics Committee, REK Sør-Øst (S-07443a), and material from still living patients was included after their written consent. Use of material from dead patients was permitted by the Ethics Committee. Animal experiments were approved by Case Western Reserve University IACUC guidelines. All steps taken for animal welfare and to minimize suffering were according to IACUC guidelines and have previously been described [22], [23].

### Cell Culture

LNCaP cells were obtained from the American Type Culture Collection (ATCC) and cultured in RPMI 1640 medium supplemented with 10% Fetal Calf Serum (FCS), 2 mM L-glutamine, 50 U/ml penicillin and 50 µg/ml streptomycin (Lonza). The human bronchial epithelial BEAS-2B cell line transformed with SV40 was a generous gift from Sten Mollerup (Department of Toxicology, National Institute of Occupational Health, Oslo, Norway) [24]. BEAS-2B cells were maintained in LHC-9 medium supplemented with 1.8 mg/ml bovine serum albumin (BSA) on plates coated with 0.01 mg/ml fibronectin and 0.03 mg/ml bovine collagen dissolved in PBS.

For both cell lines the culture media was changed every two to three days, and the cells were incubated at 37°C in a humidified 5% CO_2_, 95% air incubator. For androgen induction experiments, LNCaP cells were starved for 48 h in RPMI 1640 containing 2% charcoal treated (CT)-FCS, followed by an additional 24 h in RPMI 1640 containing 0.5% CT-FCS prior to treatment with 10^−8 ^M R1881 (synthetic androgen). R1881 was obtained from NEN.

### Xenograft Experiments

Transplantation, growth, and harvest of tumors from mice bearing CWR22 xenografts were as described previously [22], [23]. CWR22 xenografts were grown in nude mice in the presence of a sustained-release testosterone pellet. After tumor growth, mice were castrated, the testosterone pellets were removed and the regressing tumors were collected at 1, 2, or 4 weeks after castration. Animals were housed and treated according to the IACUC guidelines.

### Quantitative PCR (qPCR)

Upon harvest, total RNA was extracted from cells using Trizol® reagent (Invitrogen) according to manufacturer’s recommendations. 1–5 µg RNA was used for first-strand cDNA synthesis with the SuperScript II system (Invitrogen) and oligo-dT primers. qPCR analyses were performed on the LightCycler 480 instrument (Roche) with the LightCycler 480 SYBR Green I Master mix (Roche). A standard curve made from serial dilutions of cDNA was made to calculate the relative amount of the target and reference gene for each sample. These values were then normalized to the relative amount of the reference gene. Primers are available upon request. The differences between the groups were evaluated using a two-tailed, paired Student’s *t-*test, with *P*<0.05 being considered as significant.

### Protein Extraction and Western Analyses

Whole cell extracts were made as previously described [25], resolved by SDS-PAGE and transferred to a PVDF membrane (Bio-Rad). The blotted membrane was blocked in 10% nonfat dry milk in Tris-buffered saline (TBS) containing 0.1% Tween (TBS-Tween) for 1 h followed by incubation with primary antibody in TBS-Tween containing 5% bovine serum albumin (BSA) for 14–16 h at 4°C. Antibodies against either TCTP (1∶1000) [12], or GAPDH (1∶500) (Santa Cruz) were used. Enhanced Chemiluminescence Western blotting detection reagents (Amersham Biosciences) and analysis system were utilized for the detection of the HRP labelled proteins. For quantification, western blots were digitalized with a scanner machine (Epson Perfection V700 Photo) and the optical density was measured with the software ImageQuant TL (Amersham Biosciences). Human TCTP mouse monoclonal antibody was a generous gift from the group of del Vecchio [12].

### RNA Interference

Synthetic siRNA targeting TCTP (target sequence 5′- AACCATCACCTGCAGGAAACA-3′) was obtained from Dharmacon, while siRNA for luciferase (target sequence: 5′-AACTTACGCTGAGTACTTCGA-3′) was from Qiagen. LNCaP cells were seeded in full medium 24 h before transfection. The medium was changed to RPMI 1640 without FCS and antibiotics prior to transfection, and the cells were transfected with 100 nM siRNA per well using Oligofectamine (Invitrogen) according to the manufacturer’s protocol.

### Colony Formation Assay

LNCaP cells were transfected with siRNA as described above and plated in 10 cm plates at a density of-100000 cells/well. Colonies were grown for three weeks. LNCaP cells treated with recombinant TCTP were seeded at a density of 2500 cells per well in a six-well plate in RPMI supplemented with 10% FCS. Recombinant TCTP, GST, or vehicle control was added every two to three days. Colony formation was assessed after two weeks of growth. Cells were fixed in methanol at −20°C for 30 min, stained with 0.1% crystal violet for 20 min and then washed with MQ water. The area covered by the colonies was quantified using GeneTools software from SynGene.

### TUNEL Assay

LNCaP cells were seeded on cover-slips, starved and transfected with siRNA as described above. For thapsigargin (TG) induction, the cells were either treated with vehicle or 100 nM TG for the last 36 h of the 72 h transfection period. To detect apoptosis, a TUNEL *In Situ* Cell Death Detection Kit (Roche) was used according to the manufacturer’s instructions. Fluorescence was detected using an Axioplan2 imaging microscope (Zeiss) and pictures were taken with an AxioCam HRc camera (Zeiss). The number of TUNEL-positive cells was expressed as a percentage of the total number of cells.

### Gene Expression Profiling

Total RNA from cells treated with siRNA was isolated using the TRIzol® reagent according to the manufacturer’s instructions and was analyzed at the Norwegian Microarray Consortium (NMC), Oslo University Hospital, Oslo, Norway. The RNA was amplified using the Illumina® TotalPrep RNA Amplification kit (Illumina). 500 ng total RNA was used in the amplification and labelling reaction. The quality of the cRNA was assessed using a Bioanalyser. Biotin labelled cRNA (1.5 µg) was then used to hybridize onto Illumina Human-6 v3 Expression beadchips using the Whole-Genome gene Expression Direct Hybridization assay from Illumina. After scanning, the results were imported into Illumina BeadStudio, where the quality of each array and scan were tested.

### Generation of and Treatment with Recombinant TCTP

The open reading frame (ORF) of hTCTP was cloned into pET-28a for expression of recombinant TCTP. 0.1 mM IPTG was used to induce expression, and following harvest the pellet was resuspended in binding buffer (20 mM NaH_2_PO_4_, 500 mM NaCl, 20 mM imidazol, pH 7.4). Protease inhibitors were added and the cell suspension was sonicated at 4°C. His-tagged hTCTP was purified under native conditions using a HiTrap™ Chelating HP Column (GE Healthcare) charged with Ni^2+^-ions according to the manufacturer’s instructions. The sample was sterile filtered and diluted with binding buffer prior to application onto the column. The column was washed with binding buffer until the absorbance (280 nm) stabilized. Bound proteins were then eluted with elution buffer (20 mM NaH_2_PO_4_, 500 mM NaCl, 500 mM imidazol, pH 7.4). The purification procedure was performed at 4°C to prevent protein degradation. The eluate was dialyzed against PBS using Slide-A-Lyzer® 10K dialysis cassettes with a cut-off value of 10 kDa. To obtain recombinant GST, pGEX-4T was grown in BL21 cells, induced with 0.1 mM IPTG and purified by incubation with 50% Glutathione Sepharose 4B slurry. The beads were washed with PBS before elution (50 mM Tris-HCl, 10 mM reduced glutathione, pH 8.0). The purified proteins were run alongside Precision Plus Dual color Protein Standard, Prestained Protein Markers, Broad Range (7–175 kDa) and Unstained Protein Markers, Broad Range (2–212 kDa) (Biorad) to estimate the amount of protein.

BEAS-2B cells were treated with recombinant TCTP/GST for one h before harvest.

### Immunohistochemistry

Tissue microarrays (TMA) were prepared from radical prostatectomy specimens from patients operated at the Norwegian Radium Hospital between 1988 and 1996 and followed up after surgery. Prostate-specific antigen (PSA) measurements were performed before and after operation and at every subsequent clinical examination. Follow-up periods ranged from 2 to 176 months (mean, 73.3 months). Patients were considered to have clinically evident recurrence of disease if any of the following were present: (*a*) evidence of local recurrence (confirmed by histological biopsies or ultrasound) or (*b*) evidence of distant metastasis (detected by skeletal scintigraphy and/or magnetic resonance imaging). If a patient who suffered from relapse had postoperative serum PSA of >4 ng/ml before the date of either local recurrence or metastasis, the date of elevated PSA was set as the relapse date. H&E-stained sections were made from each selected primary tumor block (donor blocks) of paraffin-embedded material to define representative tumor regions. With the use of the tissue array instrument (Beecher Instruments), two tissue cylinders (0.6 mm in diameter) were punched from regions of the donor block. Control samples of non cancer tissue from the paraffin blocks were also taken. The Gleason score used in the analysis was the highest Gleason score in each of the prostatectomy series.

The TMAs were first de-paraffinized by xylene and serial ethanol dilutions and washed in H_2_O prior to quenching of endogenous peroxidase in 0.3% H_2_O_2_ in H_2_O for 30 min. Antigen retrieval was carried out by autoclaving at 121°C for 20 min in 0.01 M citrate buffer (pH 6.4). The BioGenex Super Sensitive Link-Label IHC Detection kit (BioGenex) was used for antigen detection. The sections were equilibrated in TBS-Tween (0.05 M Tris, pH 7.5, 0.3 M NaCl, 0.1% Tween 20) for 5 min prior to incubation overnight at 4°C with anti-human TCTP mouse monoclonal antibody diluted 1∶100 in TBS-Tween with 1% BSA. Sections were then washed with TBS-Tween prior to incubation with biotinylated secondary anti-imouse IgG (link) for 30 min at room temperature. After wash in TBS-Tween, the secondary antibody was incubated with enzyme HRP-labeled streptavidin for 30 min at room temperature. The slides were then washed in TBS-Tween and stained with DAB for 5 min and the reactions were stopped in H_2_O. Counterstain was performed by haematoxylin (DAKO) staining and the slides were mounted with DAKO Faramount aqueous mounting medium. Mouse non-immune serum was applied as negative control. Normal prostate tissue sections were used as positive controls. Intensity was scored using a 0–3 scale, corresponding to absent, weak, moderate, and intense staining, respectively. The differences between the groups were evaluated using a two-tailed, unpaired Student’s *t-*test, with *P*<0.05 being considered as significant.

### Statistics

Except for the microarray analyses, statistics were carried out using Student’s t-test where p values <0.05 were considered significant.

#### Statistical analysis of gene expression data

Statistical analysis was performed based on summary expression values for each probe and normalized by quantile normalization which results in the chips having identical intensity distribution. The data were analyzed by J-Express v2.7 software (http://www.molmine.com). Statistical Analysis of Microarrays (SAM) and Feature Subset Selection (FSS) were used for statistical analyses.

## Results

### TCTP Expression is Induced by Androgens

**Figure 1:**
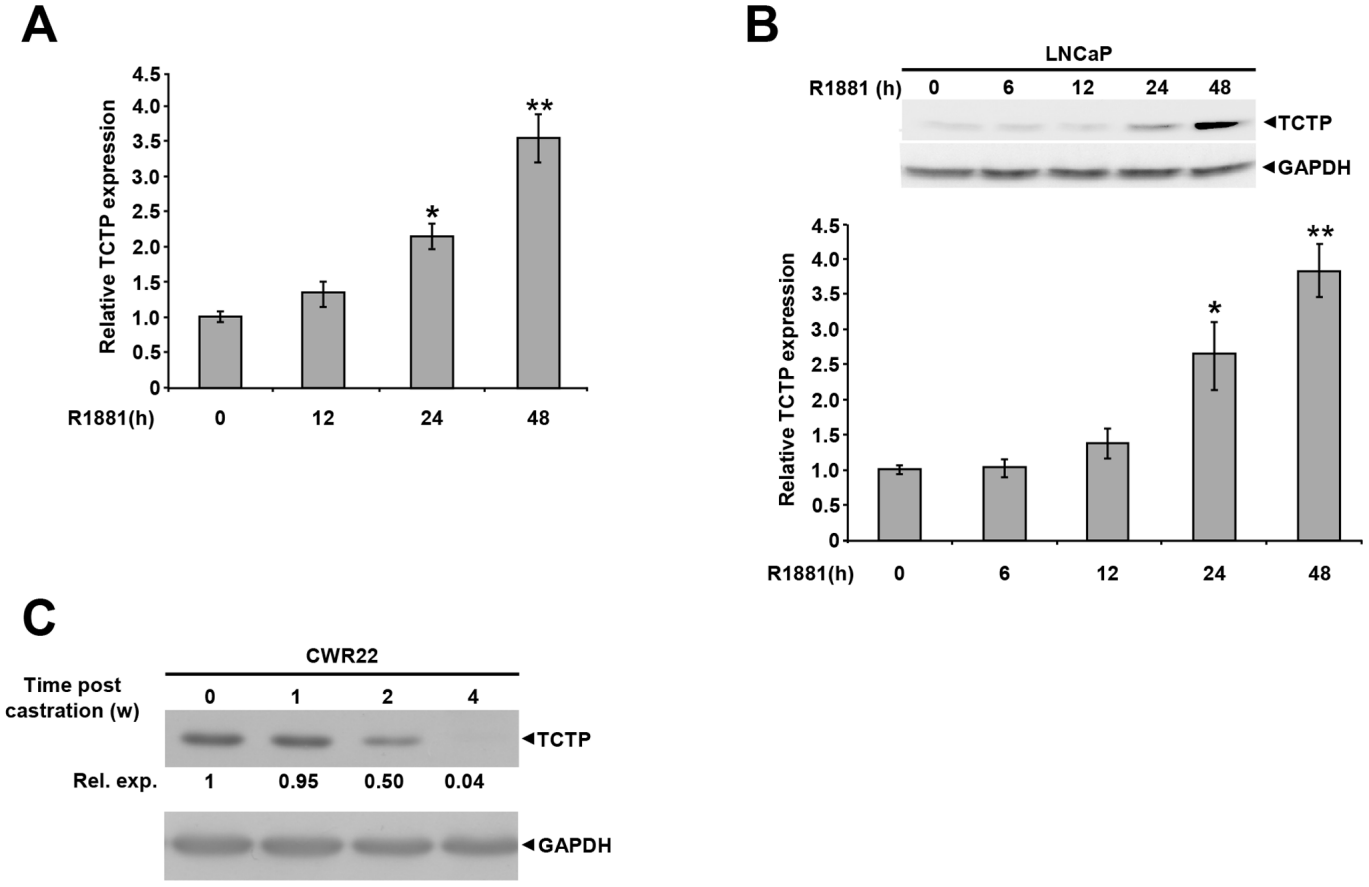
Androgen induces TCTP expression *in vitro* and *in vivo*. **A.** **** LNCaP cells were either left untreated or treated with the synthetic androgen R1881 (10^−8^ M) for the indicated times. Total RNA was isolated and qPCR analyses were performed. *TCTP* mRNA expression was normalized to *GAPDH*. The graph illustrates one representative experiment performed in triplicate with error bars indicating ±SEM, the expression levels are relative to cells treated without androgen (set to 1). The experiment was repeated more than three times **B.** Western analyses were performed on whole cell extracts made from cells treated in the absence or presence of R1881 (10^−8^ M) for the indicated time points. The expression levels were normalized to GAPDH. The values presented are relative to untreated samples (set to 1). The graph illustrates data from two experiments performed in triplicate with error bars illustrating ±SEM. **C.** CWR22 xenografts were grown in nude mice and tumor samples were collected either before (t = 0) or 1, 2 and 4 weeks after castration. Total cell extracts were made and used for western blot analyses. The figure shows one representative blot for TCTP and GAPDH, with relative values of TCTP (t = 0 set to 1) normalized to GAPDH. The differences between the groups were evaluated using two-tailed, paired Student’s t-test compared to non-treated samples, with P<0.05 being considered as significant. Significance is indicated by asterisks; one asterisk indicates P<0.05, two asterisks indicate P<0.01.

We first investigated the possible regulation of TCTP expression by androgens in the androgen responsive prostate cancer cell line LNCaP in a time course experiment. As shown in *Figures 1A *and 1B, there was an androgen-induced increase in both TCTP mRNA and protein accumulation over time, which reached approximately four-fold higher levels compared with untreated cells at 48 h. To determine if TCTP expression is also regulated by androgens *in vivo*, we used the human androgen-dependent prostate cancer xenograft model CWR22 which markedly regresses after castration [22], [23]. Western analysis of CWR22 tumors collected from mice at different time points after castration showed that the TCTP expression significantly decreased in a time-dependent manner and was nearly lost at four weeks (*Figure 1C*). These results show that TCTP expression is regulated by androgens in prostate cancer cells both *in vitro* and *in vivo*.

### Knockdown of TCTP Decreases Colony Formation of LNCaP Cells

**Figure 2:**
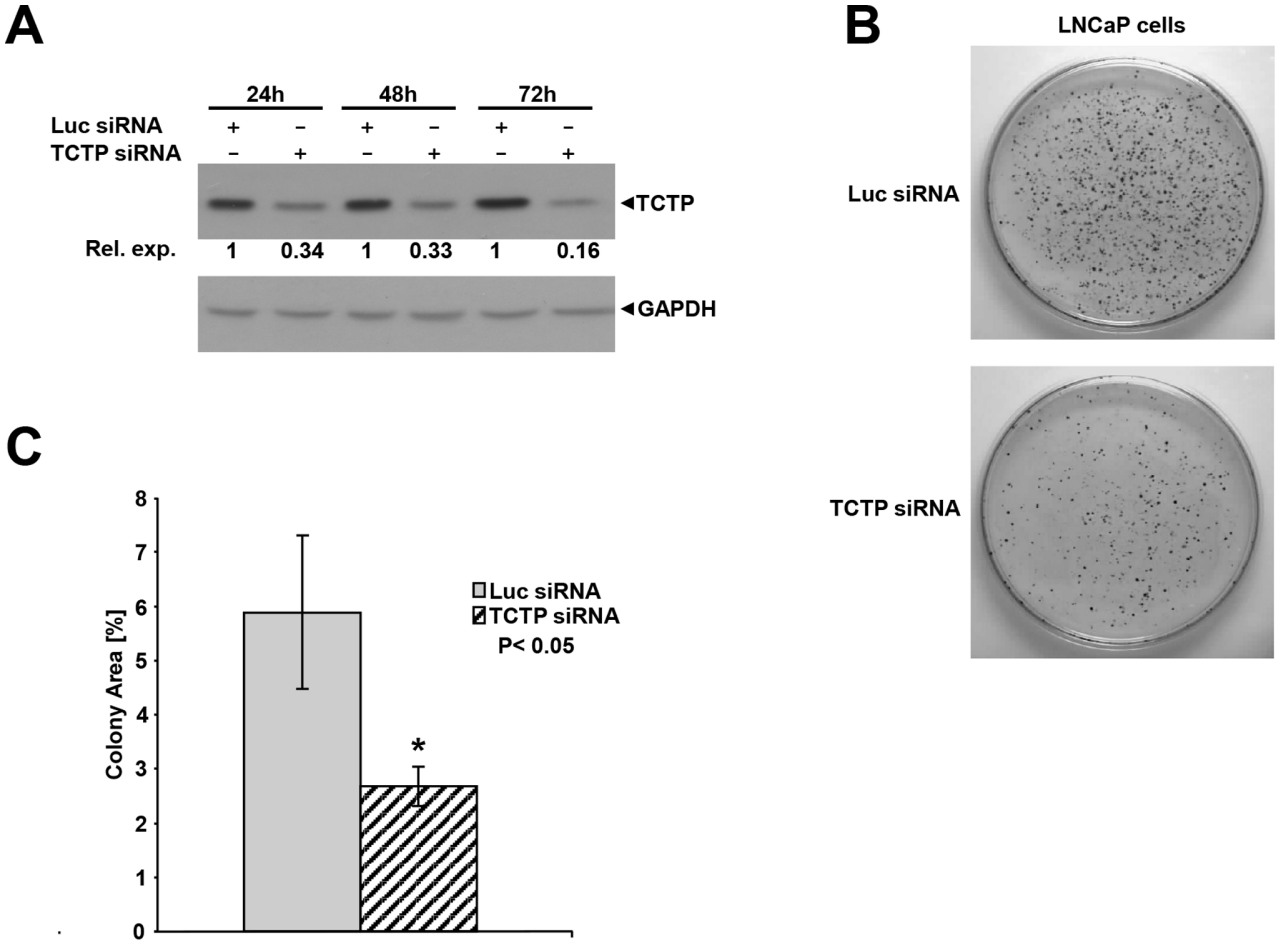
TCTP knockdown reduces colony formation of LNCaP cells. **A.** siRNA mediated knockdown of *TCTP* in LNCaP cells. LNCaP cells were transfected with siRNA (100 nM) targeting either *Luciferase (Luc)* or *TCTP*. At the indicated time points cells were harvested, protein extracts were prepared and analyzed by western analysis. Antisera raised against TCTP and GAPDH were used sequentially on the same blot. **B.** LNCaP cells were transfected with 100 nM siRNA targeting *Luc* or *TCTP*, seeded in 10 cm plates and assayed for colony formation ability by crystal violet staining after three weeks of growth. Representative pictures are shown. **C.** Quantification of colony formation of LNCaP cells after siRNA mediated knockdown of TCTP. The graph represents two experiments in triplicate. The differences between the groups were evaluated using two-tailed, paired Student’s t-test compared to Luc-siRNA treated samples, with *: P<0.05 indicating significant difference.

Androgen regulation of TCTP expression, as presented above, suggested that it may have a role in growth of prostate cancer cells. This could either be through the induction of proliferation or inhibition of apoptosis, or a combination of both. To investigate the possible role of TCTP in cell growth, its expression was inhibited in LNCaP cells by siRNA treatment and colony formation was assessed compared with control siRNA treated cells. As shown in Figure 2A, TCTP protein expression was reduced by 85% after 72 h of transfection with siRNA targeting TCTP. Colony formation of TCTP knockdown cells was decreased by 50% compared with control cells (Figures 2B and 2C). These data indicated that TCTP may have a role in LNCaP cell proliferation and/or viability.

### Down-regulation of TCTP Increases Apoptosis of LNCaP Cells

**Figure 3:**
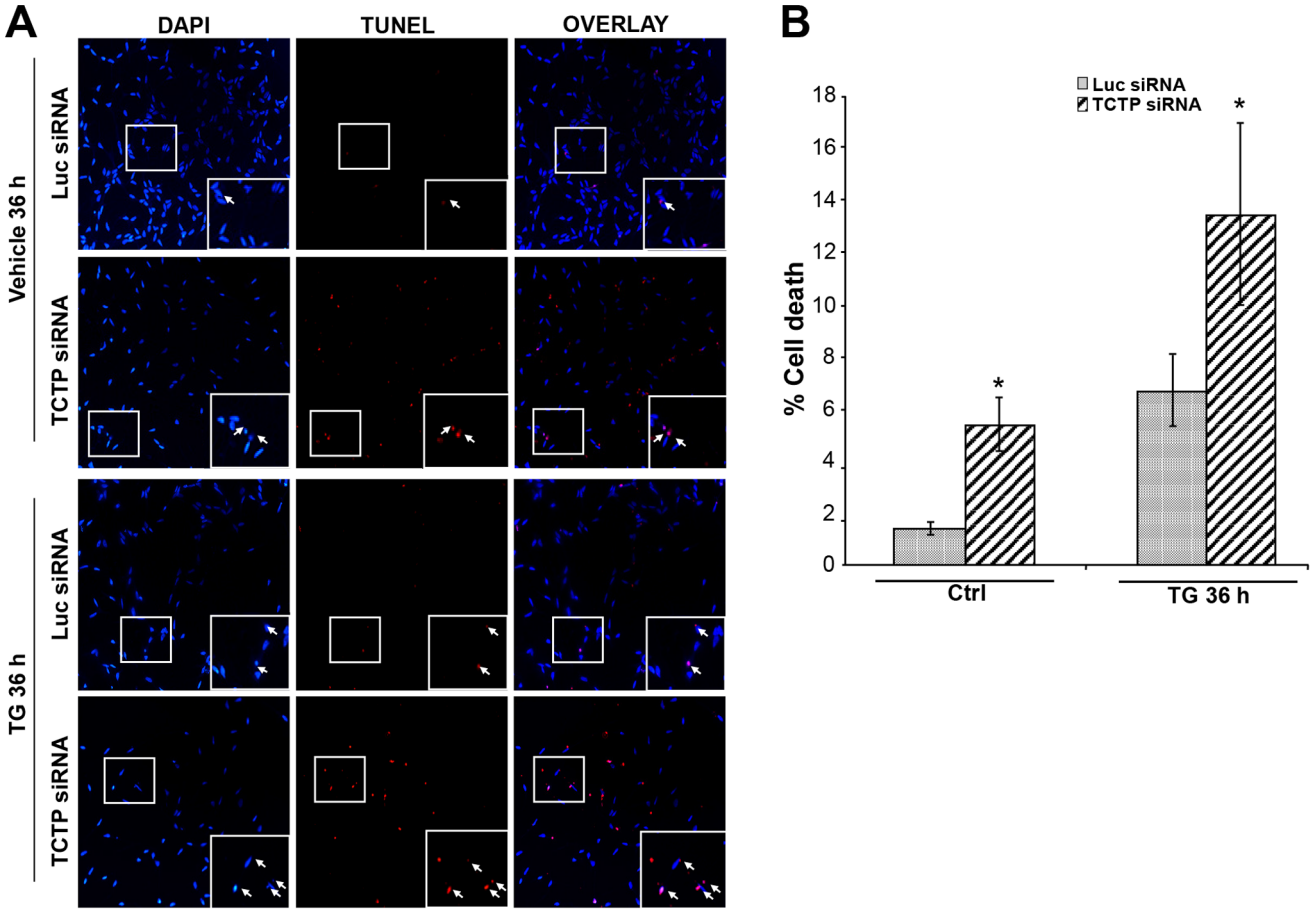
Downregulation of TCTP expression leads to increased apoptosis in LNCaP cells. LNCaP cells were cultured on coverslips and transfected with siRNA against *TCTP* or *Luciferase (Luc)* for 72 h. Apoptosis was induced by 100 nM TG for 36 h during siRNA treatment. After treatment and fixation, apoptotic cell death was detected using the TUNEL assay, cell nuclei were stained with DAPI and cells were visualized using Axioplan imaging microscope. **A.** Representative pictures of LNCaP cells transfected with either *TCTP*- or *Luc*-siRNA for 72 h and treated with DSMO or TG for 36 h during transfection. TUNEL positive cells appear as red spots. Arrows indicate apoptotic cells. **B.** Quantification of apoptosis incidence. The data show the percentage of nonviable cells after 36 h treatment with TG in cells transfected with *TCTP* or *Luc siRNA*. Columns represent the mean of three independent experiments performed in triplicate and bars represent ±SEM with *: P<0.05 indicating significant difference.

TCTP was previously reported to inhibit apoptosis in a number of cell lines [20], [26]–[28]. In addition, RNAi-mediated knockdown of TCTP in LNCaP cells activated caspase 3 and caspase 8, indicative of increased apoptosis [20], [21]. We thus investigated the possible role of TCTP on apoptosis in LNCaP cells. Previous work from our laboratory has established that Thapsigargin (TG) induces significant apoptosis after 36 h of treatment in LNCaP cells [25]. We thus determined whether TCTP may alter TG-induced apoptosis. LNCaP cells were transfected with either siRNA against TCTP or control (Luciferase) siRNA, then left untreated or exposed to TG, after which the extent of apoptosis was determined using the TUNEL assay. As shown in Figures 3A and 3B, in the absence of TG treatment, there was an approximately three-fold increase in apoptosis in TCTP knockdown cells compared with control cells. As expected, TG significantly increased apoptosis in both control or TCTP-siRNA treated cells, however, it was approximately 2.5-fold higher upon TCTP knockdown. The knockdown of *TCTP* was verified by qPCR for all experiments (data not shown). These data suggest that TCTP is involved in regulating apoptosis in prostate cancer cells.

### Down-regulation of TCTP Results in Upregulation of Immune Response Genes in LNCaP Cells

**Figure 4:**
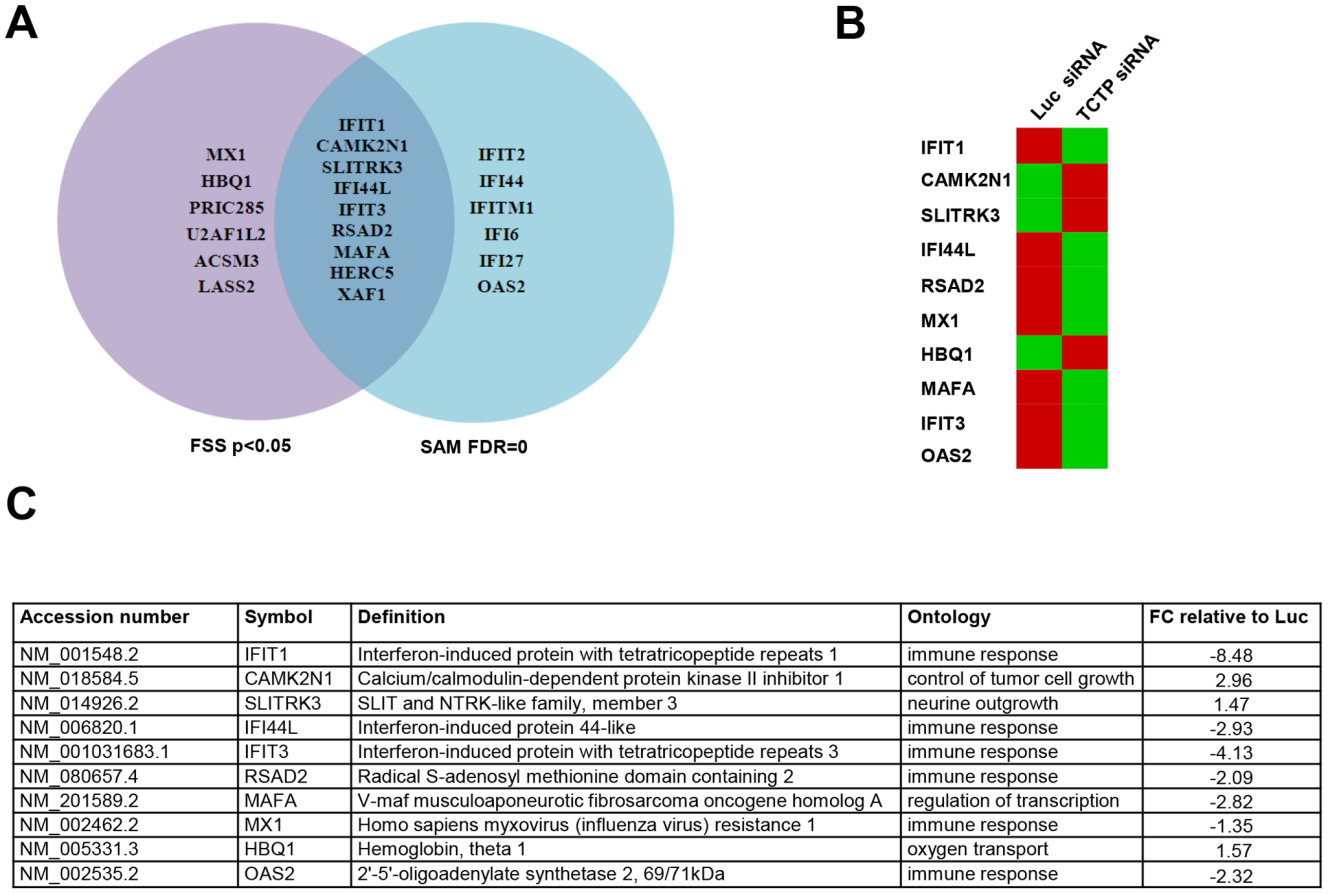
TCTP knockdown increases interferon induced gene expression. LNCaP cells were transfected with siRNA against *TCTP* or *Luciferase* for 72 h, RNA was isolated and used in global gene expression profiling as described in Materials and Methods. **A.** Venn diagram of the genes that are significantly regulated by gene expression profiling. **B.** Heatmap representation of genes up- or down-regulated in response to *TCTP* knockdown. Red and green represent up- and downregulated genes, respectively. **C.** List of the ten most significantly regulated genes with their accession numbers, definition and the ontology process for which they are implicated in. The fold change relative to *Luciferase* siRNA treated cells are indicated.

**Figure 5:**
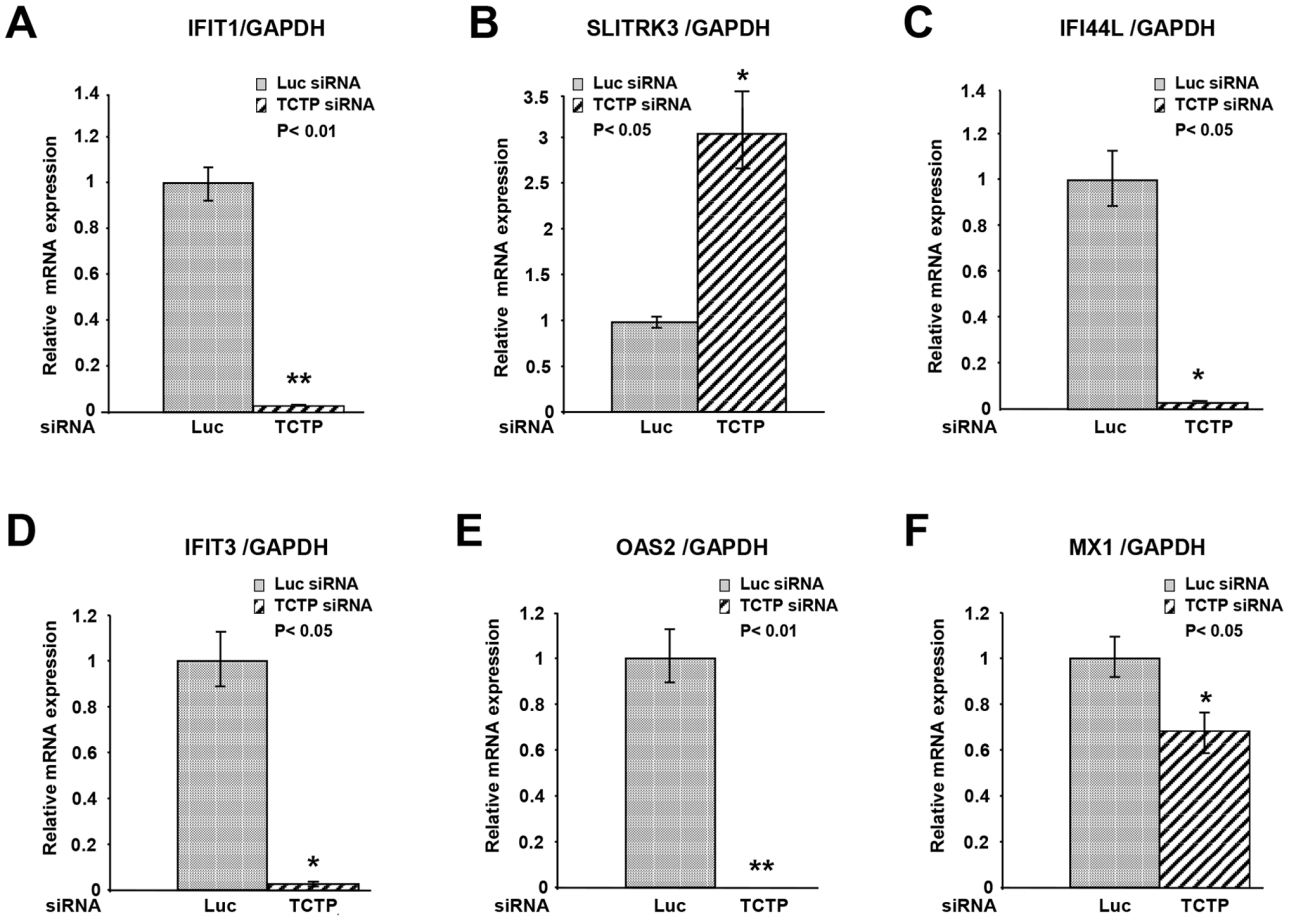
Reduction of TCTP increases interferon induced gene expression. **A–F**. qPCR was used to assess expression of genes predicted to be differentially expressed in cells tranfected with Luc-siRNA versus TCTP-siRNA. The mRNA expression was normalized to *GAPDH* and was calculated relative to Luc-siRNA samples (set to 1). Experiments were carried out in triplicate. All error bars represent ±SEM. Statistical significance was assessed using two-tailed, paired Student’s t-test with *: P<0.05 and **: P<0.01 being considered as significant.

In order to elucidate the pathways TCTP may affect in prostate cancer cells, we conducted a global gene expression profiling in TCTP knockdown cells compared with control LNCaP cells. Significant TCTP knockdown was confirmed at both mRNA and protein level (data not shown). The data were analyzed using two methods: the Statistical Analysis of Microarrays (SAM) and Feature Subset selection (FSS) tools implemented in J-Express [29]. Out of the 15 most significantly regulated genes determined by each analysis, nine were found to be significant by both methods, as illustrated in the Venn diagram in Figure 4A. Figure 4B shows up- or down-regulation of some of the genes, while a list over the most significantly regulated genes on the array, their ontology, known function and definition are depicted in Figure 4C. The majority of the genes predicted to be significantly regulated upon TCTP knockdown are involved in the interferon signaling pathway and/or immune-related responses. The expression of six of these genes (IFIT1, SLITRK3, IFI44L, IFIT3, OAS2 and MX1) was validated by qPCR (Figures 5A–F). These results imply that TCTP modulates immune responses in prostate cancer cells.

### Recombinant TCTP Induces Prostate Cancer Cell Growth

**Figure 6:**
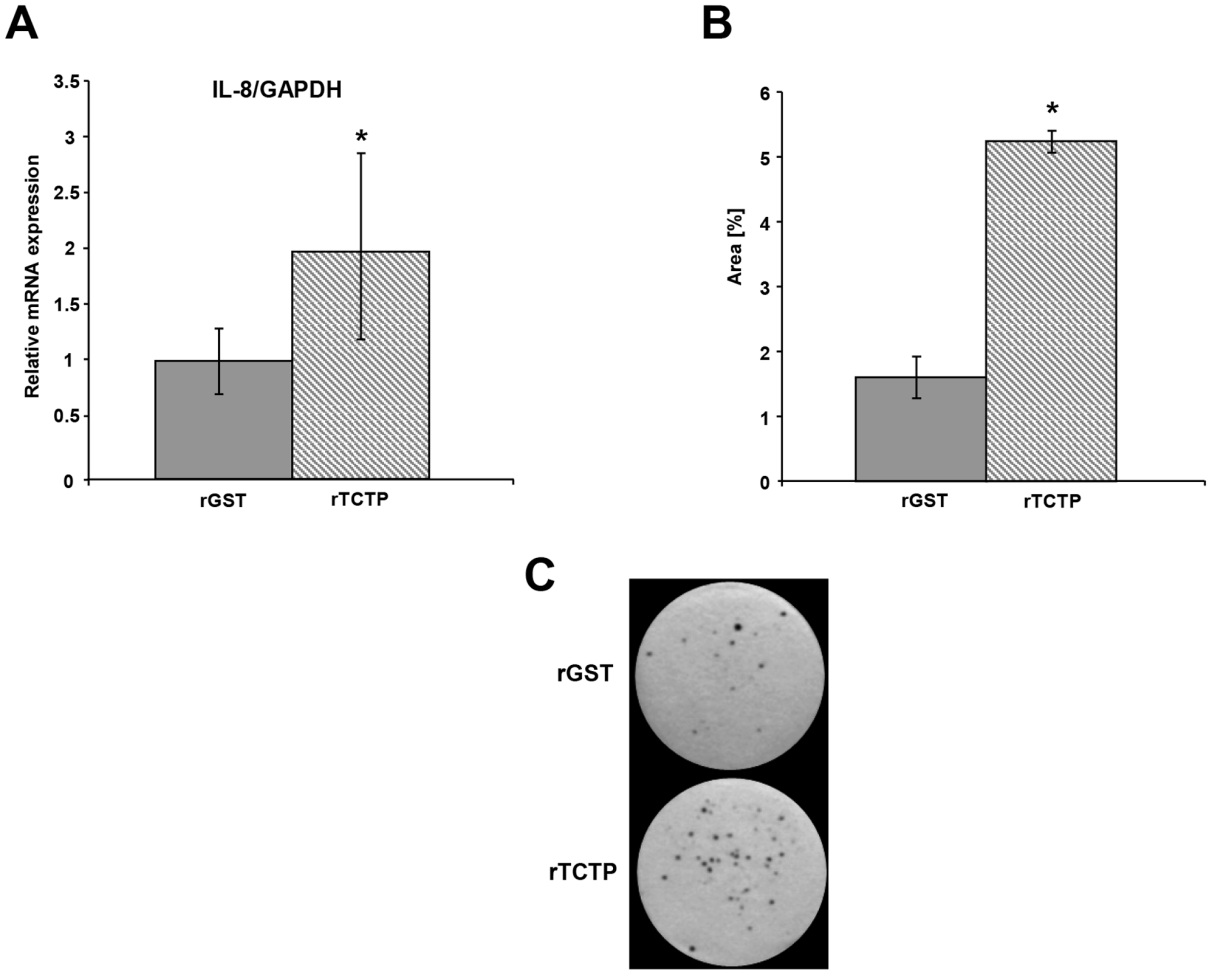
Recombinant TCTP increases colony formation of LNCaP cells. **A.** BEAS-2B cells were treated with recombinant TCTP or GST (rTCTP and rGST) to a final concentration of 1.0 µg/ml, total RNA was extracted, cDNA synthesized and qPCR performed. GAPDH was used as a reference gene and the values presented are relative to GST (set to 1). **B.** Colony formation assay in LNCaP cells treated with rTCTP or rGST. Cells were cultured in the presence of rTCTP or rGST at a final concentration of 1.0 µg/ml for two weeks and the colonies formed were visualized with 0.1% crystal violet. The area covered on each plate by the colonies was measured and represented as percentage of the total area of the plate. **C.** Two representative images are shown. The experiment was carried out in triplicate three times. Error bars represent ±SEM. Statistical significance was assessed using two-tailed, paired Student’s t-test. Significance is indicated by asterisk, P<0.05.

The secreted form of TCTP has previously been shown to induce expression of several mediators, initiate distinct signaling events and lead to an increase in cell proliferation of immune cells [30]–[32]. Since TCTP is present in prostatic fluids [12], it may have an extracellular function in prostate cancer cells. To assess this possibility, we made recombinant TCTP (rTCTP) in *E. coli* and determined its biological activity in BEAS-2B cells compared with recombinant glutathione S-transferase (rGST) as a control [33]. BEAS-2B cells were treated with rTCTP or rGST at a final concentration of 1.0 µg/ml for 1 h and *IL-8* mRNA production was determined by qPCR. *IL-8* mRNA expression was significantly increased in cells treated with rTCTP compared to cells treated with rGST (Figure 6A) indicating that the rTCTP is biologically active. LNCaP cells were then treated with 1.0 µg/ml rTCTP or rGST and a colony formation assay was performed. After two weeks in culture with continuous exposure to the recombinant proteins, cells were fixed and stained to visualize colonies. As shown in Figures 6B and 6C, there were a significantly higher number of colonies formed by the cells treated with rTCTP compared to rGST-treated cells. These data indicate that rTCTP has a proliferative/pro-survival effect on LNCaP cells and suggest a role of secreted TCTP in prostate cancer cell growth.

### TCTP Expression is Upregulated in Prostate Cancer Compared to Normal Prostate

**Figure 7:**
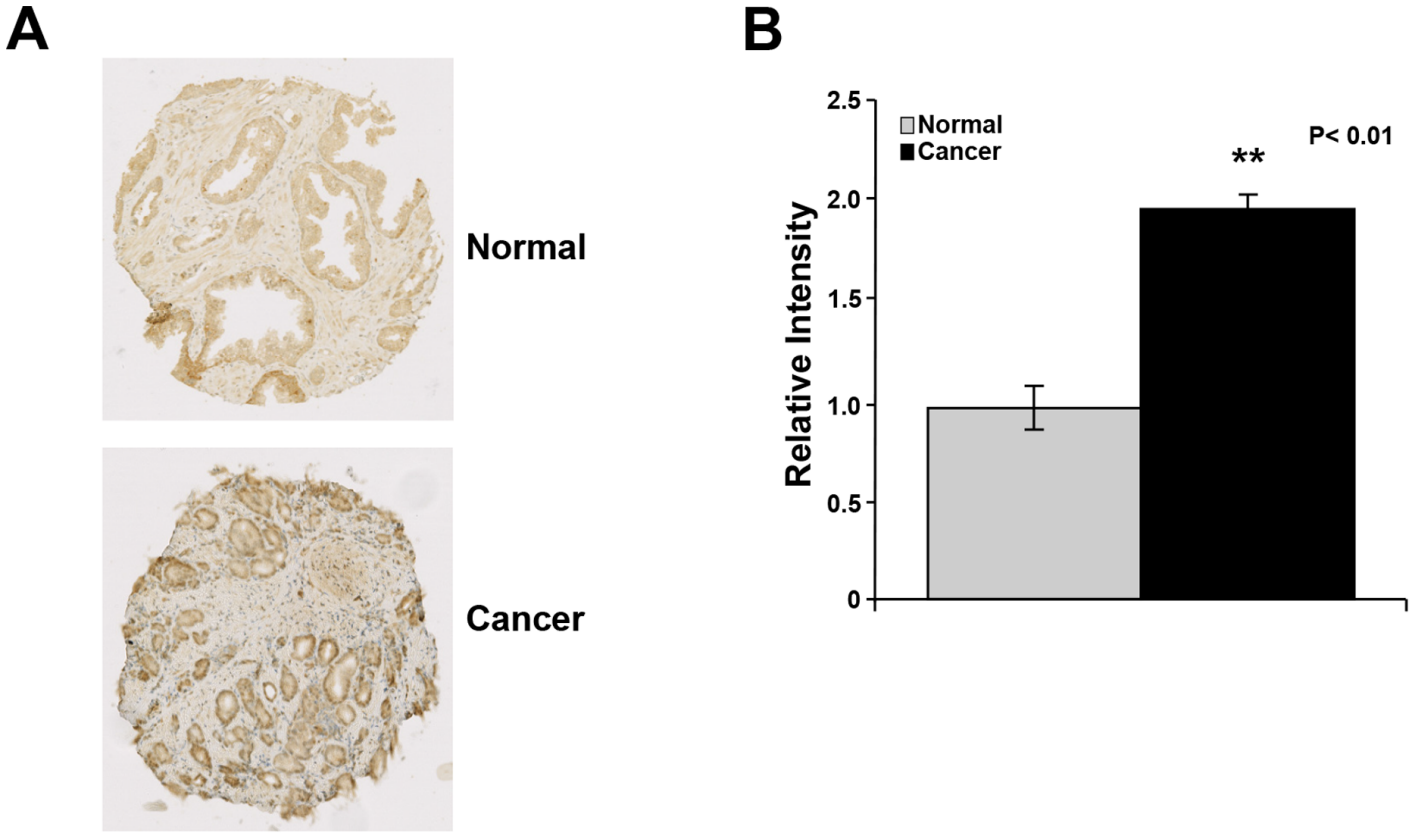
TCTP expression is increased in prostate cancer compared to normal prostate. Tissue microarrays with normal and tumor tissue from human prostate were stained with TCTP antiserum and detected by Biogenex Supersensitive Detection kit. The graph is based on 392 samples consisting of 35 benign and 357 malignant samples. The differences between the groups were evaluated using two-tailed, unpaired Student’s t-test compared to untreated samples, with P<0.05 being considered as significant. **: P<0.01.

Since TCTP is expressed in the normal prostate, as well as regulated by androgens in prostate cancer cells *in vitro* and *in vivo*, we investigated its expression on tissue microarrays (TMAs) containing normal prostate tissue as well as those representing various stages of prostate cancer using immunohistochemistry (IHC). As shown in Figure 7*A*, *T*CTP was expressed primarily in epithelial cells in both normal prostate and prostate cancer. Furthermore, its expression was significantly increased in cancer tissue compared with normal cells (Figure 7B), which indicates that TCTP may have a role in prostate cancer.

## Discussion

TCTP is a highly conserved protein in eukaryotes, from yeast to mammals, and abundantly expressed in a wide range of tissues (for a review, see 5). Several studies have underscored the multiple processes TCTP is involved in, such as immune responses, cell proliferation, tumorigenicity, and cell death [5]. The data presented here establish TCTP as an androgen regulated gene implicated in prostate cancer.

Androgen signalling and AR are critical for all phases of prostate cancer [34]. We found that TCTP is a novel androgen regulated gene whose expression is induced at both mRNA and protein level by androgens (Figures 1A and 1B). Furthermore, upon castration, the expression of TCTP dramatically declined in CWR22 androgen dependent xenografts grown in nude mice, indicating that its expression is regulated by androgens also *in vivo* (Figure 1C). Since androgens increase proliferation and inhibit apoptosis, which is the same outcome as observed in our experiments by TCTP manipulation, it is tempting to speculate that growth and viability regulating effects of androgens, at least in part, may be mediated by TCTP.

TCTP depletion in LNCaP cells significantly increased both basal levels of apoptosis, consistent with previous findings [20], as well as TG- induced apoptosis (Figure 3). These findings are consistent with a recent study that developed a TCTP antisense oligonucleotide (ASO) which inhibited the growth of PC-3 and LNCaP xenografts and significantly enhanced docetaxel activity upon systemic delivery [21]. These data open up the possibility for using TCTP knockdown in parallel with other established therapeutic approaches to increase treatment efficacy.

Gene expression profiling data indicated that approximately 60% of the differentially regulated genes are involved in the interferon (IFN) pathway (Figure 4). IFNs are a family of cytokines, named for their ability to interfere with viral infection. They mediate antiviral and antigrowth responses and are also known to modulate adaptive immune responses [35]. These findings are interesting given that TCTP was recently found to interact with NEMO, an upstream adaptor protein in the NF-κB pathway, and that NF-κB regulates transcription of inflammatory and survival genes [36]. In pilot experiments we observed that the NF-kB pathway is activated upon TCTP knockdown in LNCaP cells consistent with the notion that TCTP regulates immune responses in prostate cancer (data not shown). The IFN stimulated genes have been implicated in several cancers, including prostate cancer; however, what specific role they play in the different cancers and at what disease stage are currently unknown [37]–[42]. Interestingly, the IFN stimulated genes were not affected by androgen induction in LNCaP cells (data now shown); this could mean that TCTP may collaborate with other factors that are not regulated by androgens. Alternatively, since androgen induction of TCTP takes at least 48 h, longer time exposure to androgens may be needed to observe any effects on IFN pathway related genes. Further work is necessary for determining the possible consequence of IFN gene expression changes on PCa cell growth and viability.

The secreted form of TCTP is well-studied in immune cells, where it has been shown to function as a histamine releasing factor, induce secretion of various interleukins, initiate distinct signal transduction events, and affect cell proliferation (reviewed in [16]). Since TCTP was earlier found in prostatic fluids [12], it was suggested to have a role in prostate function and in prostate cancer; however, there have not been any studies to date which addressed the possible effect of rTCTP on prostate cancer cells. Consistent with the other data presented herein, and the function of TCTP in immune cells, we found that rTCTP increased colony formation in LNCaP cells (Figure 6). This indicates that the proliferative effects of secreted TCTP is not restricted to immune cells and is also applicable to prostate cancer cells. rTCTP has previously been implicated in the induction of distinct signal transduction pathways in immune cells [30], [32]; it is therefore of interest to investigate whether this is also the case in prostate cancer cells. Further studies elucidating the molecular mechanisms behind these results are therefore warranted.

TCTP was previously found to be expressed in normal prostate and prostate cancer cells [12], [21]; it was also found to be further increased in castration resistant prostate cancer [21]. In line with these findings, we found a significant increase in TCTP expression in a TMA representing a collection of prostate cancer samples from various cancer stages compared with benign prostate (Figure 7). These data are consistent with earlier findings where TCTP was suggested to be involved in the process of initiation and progression of castration resistant prostate cancer [21].

Taken together, our data and earlier findings suggest that TCTP expression is relevant for human prostate cancer. TCTP may have a unique role in regulating inflammation and carcinogenesis processes thought to be tightly linked, making it a potential biomarker and a therapeutic target in prostate cancer.
