# Correction: TCTP Is an Androgen-Regulated Gene Implicated in Prostate Cancer

**DOI:** 10.1371/journal.pone.0128727

**Published:** 2015-05-14

**Authors:** Mari Kaarbø, Margrethe L. Storm, Su Qu, Håkon Wæhre, Bjørn Risberg, Håvard E. Danielsen, Fahri Saatcioglu

There is an error in the first sentence of the “RNA interference” subsection of the Materials and Methods. The correct sentence is: Synthetic siRNA targeting TCTP (target sequence 5’- CCATCACCTGCAGGAAACA-3’) was obtained from Dharmacon, while siRNA for luciferase (target sequence: 5’-AACTTACGCTGAGTACTTCGA-3’) was from Qiagen.

There is an error in the title of the fourth subsection of the Results. The correct title is: Down-regulation of TCTP Results in Down-regulation of Immune Response Genes in LNCaP Cells.

There is an error in the legend for Fig 4. Please see the complete, corrected Fig 4 here.

**Fig 4:**
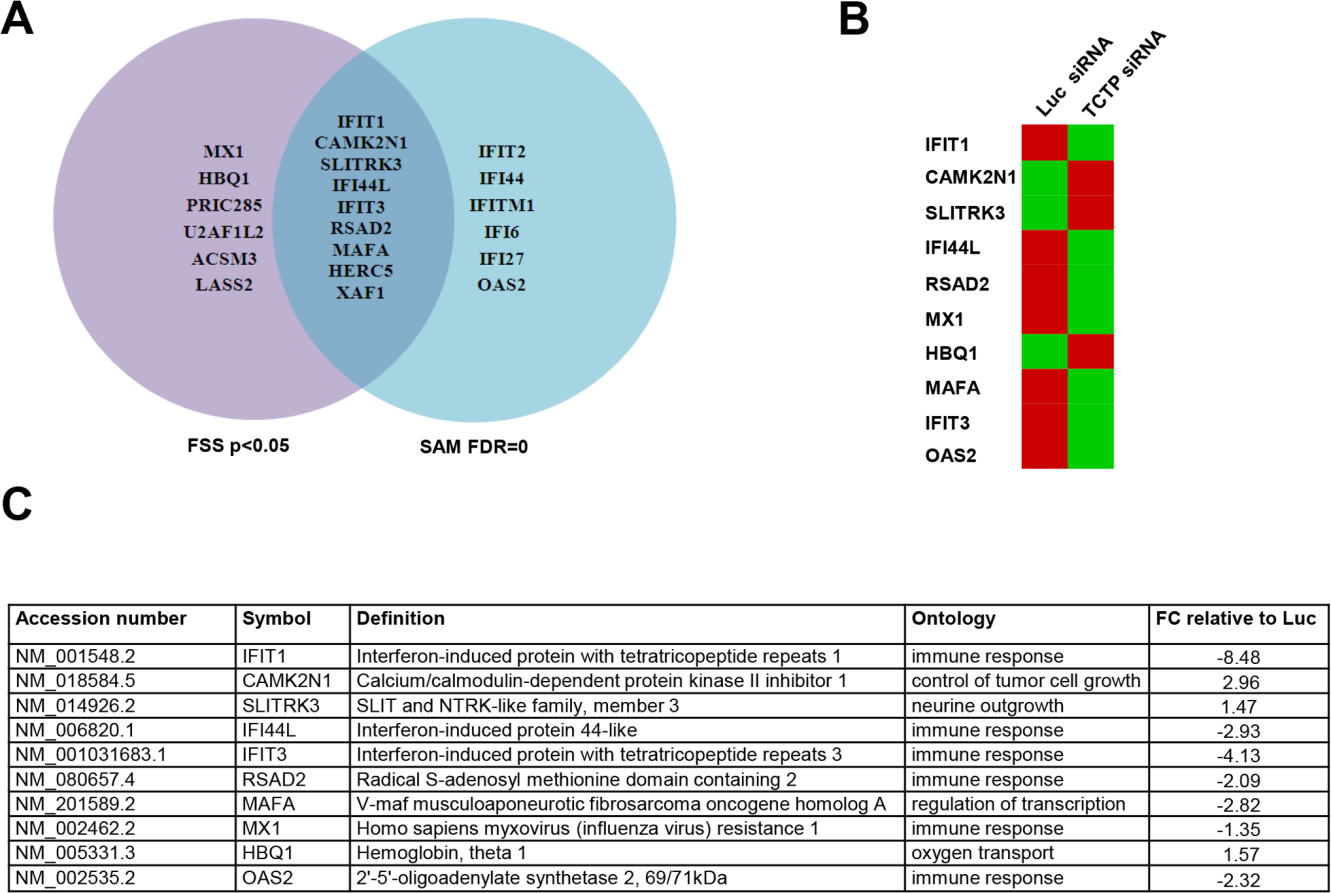
TCTP knockdown decreases interferon induced gene expression. LNCaP cells were transfected with siRNA against *TCTP* or *Luciferase* for 72 h, RNA was isolated and used in global gene expression profiling as described in Materials and Methods. **A.** Venn diagram of the genes that are significantly regulated by gene expression profiling. **B.** Heatmap representation of genes up- or down-regulated in response to *TCTP* knockdown. Red and green represent up- and downregulated genes, respectively. **C.** List of the ten most significantly regulated genes with their accession numbers, definition and the ontology process for which they are implicated in. The fold change relative to *Luciferase* siRNA treated cells are indicated.

There is an error in the legend for Fig 5. Please see the complete, corrected Fig 5 here.

**Fig 5:**
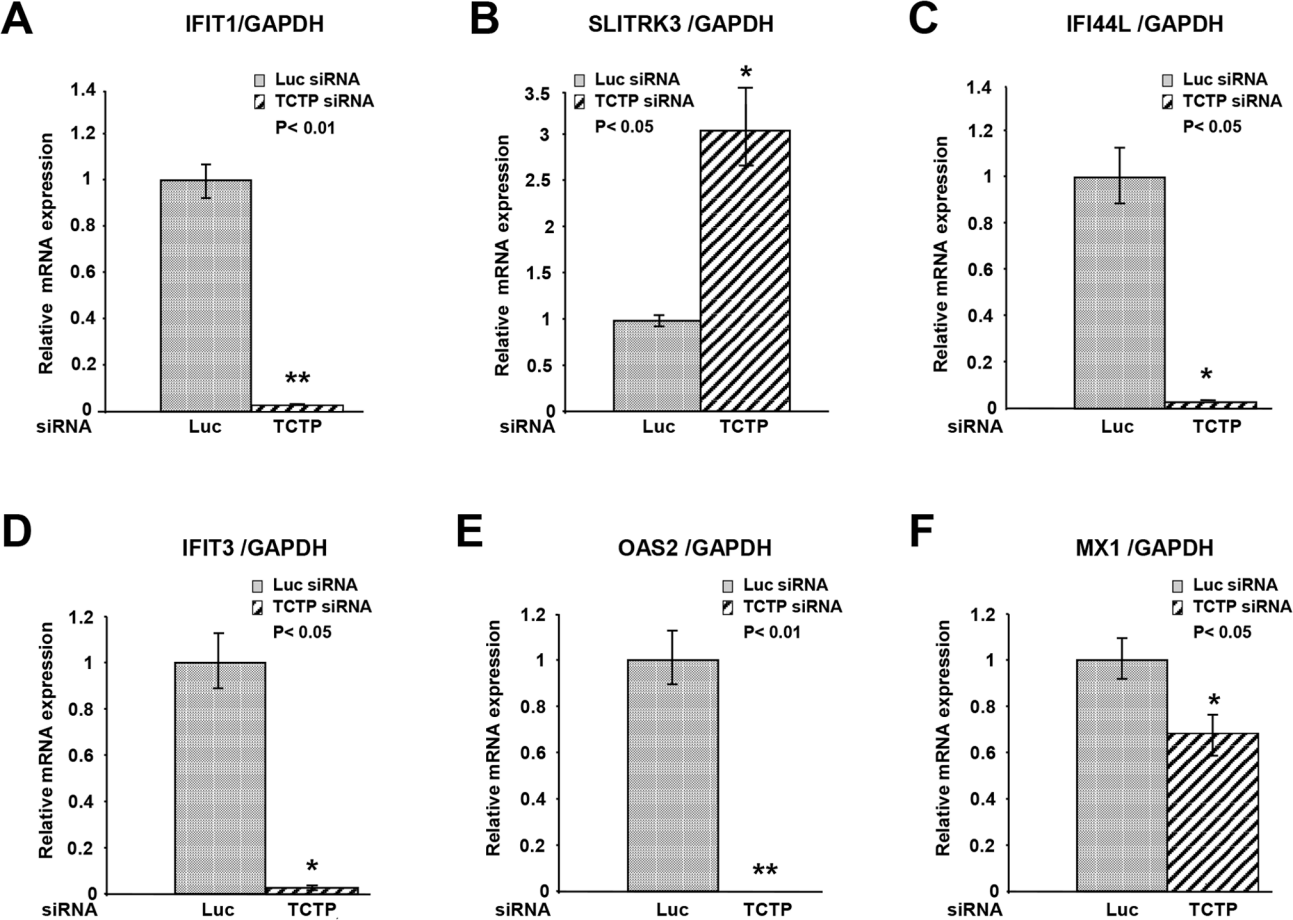
Reduction of TCTP decreases interferon induced gene expression. **A-F**. qPCR was used to assess expression of genes predicted to be differentially expressed in cells tranfected with Luc-siRNA versus TCTP-siRNA. The mRNA expression was normalized to *GAPDH* and was calculated relative to Luc-siRNA samples (set to 1). Experiments were carried out in triplicate. All error bars represent ±SEM. Statistical significance was assessed using two-tailed, paired Student’s t-test with *: P<0.05 and **: P<0.01 being considered as significant.
